# Triplet Delivery following Unilateral Twin Salpingocyesis

**DOI:** 10.1155/2015/512845

**Published:** 2015-04-02

**Authors:** Johnson O. Komolafe, Rasaq A. Akindele, Adeniyi O. Fasanu, Callistus A. Akinleye, Taiwo O. Akinbile, Monisayo O. Komolafe, Muyiwa O. Oguntunde

**Affiliations:** ^1^Department of Obstetrics & Gynaecology, College of Health Sciences, PMB 5000, Isale-Osun, Osogbo, Osun State, Nigeria; ^2^Ayomide Women's Health Specialist Hospital, Osogbo, Osun State, Nigeria; ^3^Department of Community Medicine, College of Health Sciences, PMB 5000, Isale-Osun, Osogbo, Osun State, Nigeria; ^4^Department of Obstetrics & Gynaecology, Lautech Teaching Hospital, Osogbo, Nigeria; ^5^Renal Unit, Nursing Department, Lautech Teaching Hospital, Osogbo, Osun State, Nigeria

## Abstract

We present the case of a 36-year-old woman with primary infertility of six-year duration who had IVF/ICSI on account of male factor infertility. Transvaginal scanning done on the 30th day following embryo transfer revealed an empty uterine cavity with two gestational sacs containing active fetal echoes in the right adnexum. Patient reluctantly had right salpingectomy via open laparatomy. The patient had repeat embryo transfer eleven months afterwards that culminated in the delivery of living twins with a fetal papyraceous.

## 1. Introduction

Ectopic pregnancy (salpingocyesis) following IVF cycle is not a rare occurrence with a range from 1.46% to 4.9% [1, 2]. Heterotopic pregnancy in IVF treatment is not as common as salpingocyesis with an incidence of 0.3% to 1% [3]. Common documented risk factors for salpingocyesis are tubal factor infertility, previous ectopic pregnancy, previous surgery for endometriosis, and previous myomectomy [1, 3]. However, having double (twin) gestational sacs outside the regular uterus is not common. None has been recorded following natural conception to the best of our knowledge. We document the second case of twin ectopic gestation following IVF with a peculiar emotional challenge of refusal of treatment by a woman who had waited for six years to ever achieve any pregnancy. The initial set back, however, gave way to triplet intrauterine pregnancy leading to delivery of living twins.

## 2. Case Report

The patient was a 36-year-old nullipara referred to our fertility unit by a gynaecologist on account of 6-year history of inability to achieve pregnancy. Details of assessment prior to referral were not made available but summary revealed primary infertility due to male factor infertility. She had ovulatory cycles; tubal factor was not investigated because of no apparent risk factor. She had undergone four failed intrauterine inseminations with husband semen.

Assessment revealed that uterus was retroverted, and day 3 FSH value is affirmed to be 9.9 I.U/L. Semen volume was 2.2 mLs, highly viscous with sperm concentration of 5 million/mL, total sperm number per ejaculate being 11 million, progressive motile sperm of 28%, and normal sperm forms of 11%. Based on duration of infertility with severe male factor couple was counseled for IVF-ICSI (in vitro fertilization with intracytoplasmic sperm injection cycle). She had a long protocol agonist cycle using daily buserelin injection and highly purified FSH/LH (Menopur). Four grade 1 embryos (three 6-cell and one 4-cell) were transferred into the uterus on day 2 of embryo life using Wallace soft embryo transfer catheter. Introducer was used and embryo transfer time was approximately 4 minutes.

**Figure 1:**
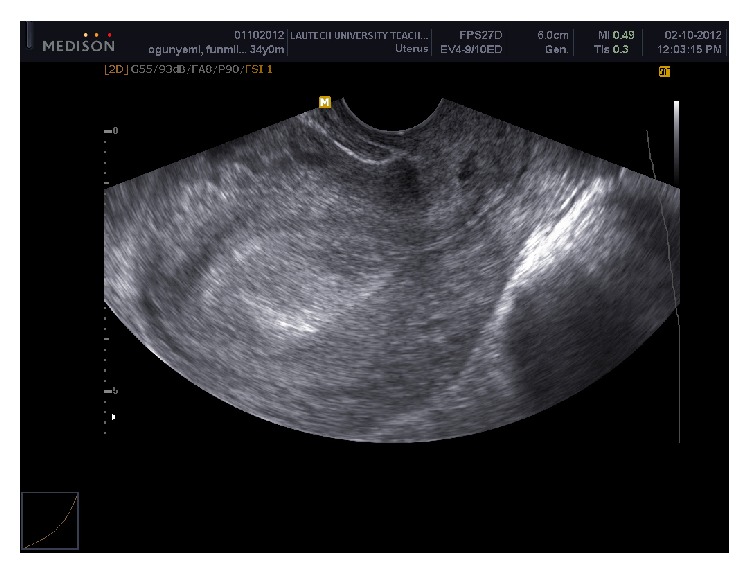
Thick endometrial lining with absent gestational sac.

**Figure 2:**
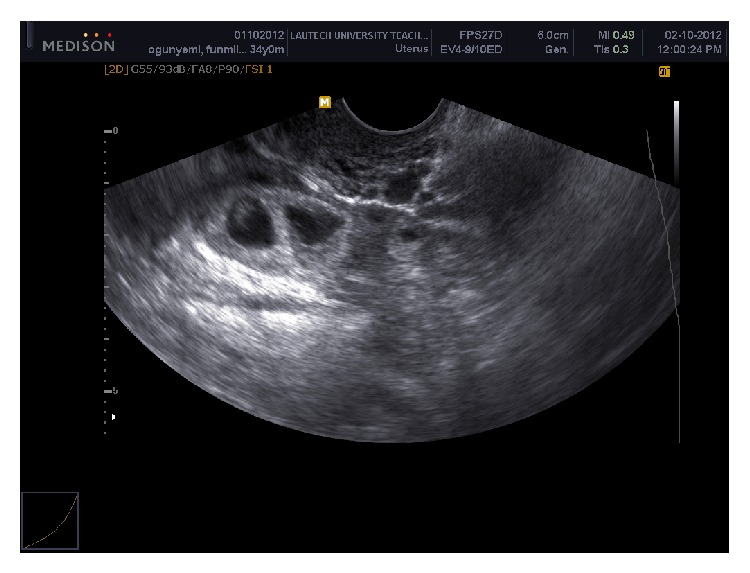
Twin gestational sacs in right fallopian tube.

Urine pregnancy test was positive on day 20 of embryo life. TVS done on day 33 of embryo life revealed uterus devoid of gestational sac with thickness of 20 mm endometrial lining (Figure 1) with double gestational sacs with viable fetal poles (Figure 2) in right adnexum and normal right ovary. Couple was counseled on possible modes of treatment. The woman initially refused any modality that would threaten continuity of pregnancy though ectopic in location despite possible risk to her life. It was difficult to accept losing these pregnancies which she had waited 6 years to achieve. After 48 hours of diagnosis she gave consent to laparatomy when one of the two sacs started to leak blood and she developed colicky abdominal pains. She requested for transfer of unruptured gestation into her uterus at laparatomy if it was possible. At operation, findings were hemoperitoneum of 250 mLs, uterus was bulky and bound down by adhesions, left tube was not visualized at all, and left ovary was seen with difficulty. Right tube looked deformed with ectopic gestation in ampullary region and leaking spot was 5 cm short of fimbria. The patient had partial right salpingectomy because adhesions prevented total salpingectomy. She was discharged home on postoperative day 3 in stable condition.

She represented for another cycle ten months following the salpingectomy. Using the same protocol above, she had three embryos (four-cell grade 1, two-cell grade 1, and one-cell embryos) transferred on day two of embryo life. She became pregnant and ultrasound diagnosis of triplet gestation was made. One of the fetuses was noticed to have died at gestational age of twenty-four weeks. She subsequently had elective abdominal delivery of living twins (female 2.7 kg and male 2.4 kg) and a fetal papyraceous at 37 weeks of gestation.

## 3. Discussion

This is the second case of twin ectopic gestation following IVF treatment documented as far as extensive search could reveal. The first was in a 37-year-old woman described by Göker et al. in Ege University, Turkey [4]. Risk factors which could have predisposed our client to ectopic gestation were undiagnosed tubal factor infertility and pelvic adhesions. Other factors were transfer of fresh embryo in a stimulated cycle as opposed to transfer of thawed frozen embryo in a natural cycle, transfer of more than one embryo & difficult transfer as evidenced by the use of an introducer. The use of introducer could have led to uterine contractions that forced the embryo into the diseased tube [1–3]. Another apparent factor might be the day 2 transfer. Strandell et al. in their study saw no ectopic gestation after day 3 transfer attributing it to possible effect of progesterone protecting against ectopic gestation when used for early luteal support [3]. Another explanation to Strandell et al.'s findings may be that day 3 embryos which might be anything from 12-cell upwards are unlikely to be able to pass through the tubal ostium as compared to day 2 embryo.

Management of this client should have been by operative laparoscopy or interventional intracardiac injection of potassium chloride. At the time of presentation, our unit did not have facility for operative laparoscopy but had facility and expertise for intracardiac injection of potassium chloride. The client in question did not give consent for medical feticide until she started leaking from one of the ectopic gestations with accompanying abdominal pains. She asked for possible transplantation of the unruptured gestation into the uterus. No record of such case is found in the literature. Theoretically, the ampullary portion containing the gestation can be transplanted into the uterine cavity through a hole made in the wall of the uterus laparoscopically. It can be argued that if the gestation now ruptures, it can secondarily implant in the endometrium. Theoretical issues would be risk of rupture from the point of transplantation through myometrium and pain from uterine contractions to possibly expel the apparent foreign body. This patient would not have been a good candidate as her right tube could not be mobilised due to adhesions.

She waited for almost a year before she could come for repeat IVF treatment due to financial constraint. She eventually had a twin delivery of living fetuses with a third fetal papyraceous. The fetal demise probably occurred due to congenital abnormality traceable to the embryo resulting from one-cell transfer at 48 hours after egg retrieval. It meant that the fertilised egg eventually cleaved though lately being a pointer to possible chromosomal abnormality especially polyploidy and mosaicism [5, 6]. The one-cell embryo should not have been transferred.

Could this patient have had single elective blastocyst transfer [7]? Yes but the chances of having an embryo that gets to day 5 in this particular cycle are too slim considering total number of embryos available and coupled with the fact that the facility does not offer preimplantation diagnosis of embryos at the moment, we consider it makes better sense to put 2 or three embryos to have the chance of having one normal embryo to implant. Nature usually will disallow implantation/continuity of abnormal embryo which constitutes between 52% and 62% of all embryos [5, 6].
